# Brace Treatment Is Associated with Lower Curve Progression in Moderate Adolescent Idiopathic Scoliosis, While Psychosocial Outcomes Are Driven by Depressive Symptoms and Curve Progression

**DOI:** 10.3390/jcm15062375

**Published:** 2026-03-20

**Authors:** Ahmet Keskin, Niyazi Igde, Mustafa Serpi, Gorkem Kayis, Huseyin Sina Coskun, Mehmet Akif Kaygusuz

**Affiliations:** 1 Department of Orthopedics and Traumatology, Metin Sabanci Baltalimanı Bone Diseases Training and Research Hospital, Istanbul 34470, Turkey; mustafaserpi@gmail.com (M.S.); drgorkemkayis@gmail.com (G.K.); makifkaygusuz@gmail.com (M.A.K.); 2 Department of Orthopedics and Traumatology, Levent Hospital, Istanbul 34415, Turkey; niyazi.igde@gmail.com; 3 Department of Orthopedics and Traumatology, Ondokuz Mayıs University, Samsun 55200, Turkey; sina.coskun@hotmail.com

**Keywords:** adolescent idiopathic scoliosis, brace treatment, spinopelvic balance, quality of life, body image

## Abstract

**Background:** The impact of thoracolumbosacral orthosis (TLSO) bracing on sagittal spinopelvic alignment and psychosocial outcomes in adolescent idiopathic scoliosis (AIS) remains debated. **Methods:** This retrospective comparative study included 120 girls (10–18 years) with AIS (baseline Cobb angle 20–40°) and skeletal immaturity (Risser 0–1). Patients were managed with a thoracolumbosacral orthosis (TLSO) brace (*n* = 60) or observation alone (*n* = 60). Standing posteroanterior and lateral full-spine radiographs were obtained at baseline and at 24 ± 6 months; follow-up radiographs were acquired out of brace after a standardized 48 h brace-free interval. They were used to measure coronal and sagittal spinopelvic parameters. Patient-reported outcomes included the Scoliosis Research Society-22r (SRS-22r), Pediatric Quality of Life Inventory (PedsQL), Spinal Appearance Questionnaire (SAQ), Trunk Appearance Perception Scale (TAPS), and Beck Depression Inventory (BDI). The primary endpoint was curve progression (≥5° increase or exceeding 40°) at 24 months. Multivariable regression was used to adjust for baseline Cobb angle and maturity. Mean follow-up was 24 ± 6 months. **Results:** Mean Cobb change was +1.2° in the brace group vs. +7.3° in the observation group (group × time interaction *p* < 0.001). Progression (≥5°) occurred in 15% vs. 45%, respectively, and 18% of observed patients exceeded 40° (risk ratio 0.33, 95% CI 0.17–0.65; number needed to treat 4, 95% CI 3–7). Sagittal spinopelvic parameters showed no significant group-by-time interaction. No significant between-group differences were observed in SRS-22r, PedsQL, SAQ, TAPS, or BDI at baseline or follow-up. Patients with curve progression exhibited worse appearance-related scores. In multivariable analysis, depressive symptoms were the strongest determinant of PedsQL (β = −0.55, *p* < 0.001). **Conclusions:** Brace treatment was associated with reduced curve progression in girls with moderate AIS. Over approximately two years of follow-up, we did not observe clinically relevant between-group differences in sagittal spinopelvic alignment or psychosocial patient-reported outcomes. Given the retrospective, non-randomized design and self-reported adherence, psychosocial findings should be interpreted cautiously and require confirmation in prospective, objectively monitored, psychologically informed bracing studies.

## 1. Introduction

Adolescent Idiopathic Scoliosis (AIS) is the most common spinal deformity in adolescence, and its prevalence is between 0.5 and 5% [1]. Both the incidence and the risk of progression are higher in girls than in boys [2]. The standard treatment for growing patients with moderate curvature (20–40° Cobb angle) is to use a brace for at least 18 h a day to stop curvature progression [3,4,5]. The BrAIST study proved the effectiveness of the brace in preventing curvature progression with a dose–response relationship [6]. However, the success of treatment should be evaluated not only with radiological but also with psychosocial results [7]. In the cohort study of Cheung et al., it was shown that the quality of life of patients using braces was lower than that of the observation group, but this difference disappeared after treatment [8].

However, radiographic outcomes alone do not capture the full clinical impact of AIS management. Adolescence is a vulnerable period for self-image and psychological well-being, and both scoliosis and brace wear have been associated with body image concerns, reduced self-esteem, and impaired health-related quality of life in some cohorts, whereas other studies report minimal or transient psychosocial effects when brace treatment is appropriately supported [9,10,11].

AIS is a three-dimensional deformity, and sagittal alignment and spinopelvic parameters are increasingly recognized as clinically relevant [12,13]. Adaptive pelvic mechanisms may accompany thoracic hypokyphosis in AIS, and braces may influence the sagittal spinal profile [14,15]. Importantly, psychosocial outcomes appear to be influenced not only by coronal curve magnitude but also by perceived deformity and depressive symptoms [10,12,16]. Nonetheless, longitudinal comparative data simultaneously addressing curve progression, sagittal spinopelvic alignment, and validated multidomain psychosocial patient-reported outcomes in braced versus observed patients remain limited.

Therefore, this study aimed to compare coronal and sagittal radiographic parameters together with quality of life, body image, and depressive symptoms in skeletally immature girls with moderate AIS managed with TLSO bracing versus observation. We additionally explored associations between curve progression, body image measures, depressive symptoms, and quality of life, and evaluated predictors of curve progression and quality of life using regression models.

## 2. Material and Method

### 2.1. Study Design and Setting

This retrospective comparative cohort study was conducted at the University of Health Sciences Baltalimanı Bone Diseases Training and Research Hospital and included patients evaluated between January 2020 and May 2023. The study analyzed routinely collected clinical, radiographic, and questionnaire data from outpatient follow-up visits.

### 2.2. Participants

Female patients aged 10–18 years were eligible if they had: a diagnosis of adolescent idiopathic scoliosis; a baseline major curve Cobb angle between 20° and 40°; skeletal immaturity defined as Risser grade 0–1; and no prior surgical treatment for scoliosis. Exclusion criteria were neuromuscular scoliosis, congenital scoliosis, and other secondary causes of scoliosis.

### 2.3. Treatment Groups and Exposure Definition

Patients were managed with either TLSO bracing or observation alone according to routine clinical decision-making and shared decision-making with patients and families. In general, bracing was recommended for skeletally immature patients with moderate curves in line with established clinical practice [3,4]. Observation was selected when bracing was deferred based on clinical judgment and patient/family preference within the same moderate-curve range. Treatment allocation was not randomized.

### 2.4. Brace Protocol and Adherence Assessment

Patients in the bracing group were prescribed a TLSO and were advised to wear the brace for 18–23 h/day. The mean brace treatment duration was approximately 20 months. Brace adherence was assessed from outpatient records based on patient/parent reports documented during follow-up visits (free-text clinician note). For exploratory analyses, adherence was categorized as low (<8 h/day), intermediate (8–16 h/day), and high (≥16 h/day). Because adherence was based on a patient/parent report documented in routine outpatient notes, it is subject to recall and social-desirability bias and may overestimate true brace wear; therefore, adherence results were considered descriptive.

### 2.5. Radiographic Measurements

Standing posteroanterior and lateral full-spine radiographs were obtained at baseline and at final follow-up (24 ± 6 months). Radiographs were acquired in a standardized standing position. Control radiographs in braced patients were obtained out of the brace after a standardized brace-free interval of 48 h to minimize immediate in-brace effects on alignment measurements.

Coronal plane assessment included measurement of the major curve Cobb angle (degrees). Sagittal and spinopelvic parameters included pelvic incidence (PI), pelvic tilt (PT), sacral slope (SS), thoracic kyphosis (TK), lumbar lordosis (LL), spinopelvic angle (SPA), and spinosacral angle (SSA). Pelvic incidence was treated as an anatomical parameter and is reported at baseline.

Radiographic measurements were performed independently by two orthopedic surgeons using digital measurement tools on standing anteroposterior and lateral full-spine radiographs. Prior to measurement, all images were anonymized (patient identifiers removed) and presented in a randomized order to minimize expectation bias; the mean of the two observers’ measurements was used for analysis. Curve progression was defined using a 5-degree threshold, which is commonly used in clinical studies to exceed typical measurement variability in Cobb angle assessment.

### 2.6. Patient-Reported Outcomes Measures

Validated Turkish versions of the following scores were administered during routine outpatient visits at baseline and follow-up: Scoliosis Research Society-22r (SRS-22r) for scoliosis-specific health-related quality of life; Pediatric Quality of Life Inventory (PedsQL) for generic quality of life; Spinal Appearance Questionnaire (SAQ) for appearance-related concerns; Trunk Appearance Perception Scale (TAPS) for trunk appearance perception; and Beck Depression Inventory (BDI) for depressive symptoms.

### 2.7. Outcomes

The primary outcome was defined as curve progression at follow-up, defined as an increase in Cobb angle of ≥5° from baseline and/or exceeding 40° at follow-up. Secondary outcomes were defined as changes in sagittal spinopelvic parameters and changes in psychosocial outcomes (SRS-22r, PedsQL, SAQ, TAPS, and BDI) over time. Exploratory outcomes were defined as associations among radiographic measures, body image, depressive symptoms, and quality of life, and the relationship between brace adherence and treatment satisfaction.

### 2.8. Statistical Analysis

Statistical analyses were performed using IBM SPSS version 27.0 (IBM Corp., Armonk, NY, USA). Continuous variables are presented as mean ± standard deviation (SD), and categorical variables as *n* (%). Between-group comparisons used independent-samples *t* tests or Mann–Whitney U tests as appropriate; within-group changes used paired t tests.

Group (brace vs. observation) and time (baseline vs. follow-up) effects were assessed using a two-way mixed model (group × time) ANOVA for continuous outcomes. Effect sizes for repeated-measures models are presented as partial eta-squared (partial η^2^), and standardized effect sizes for between-group comparisons are presented as Cohen’s d where appropriate. For the primary endpoint, we additionally report risk ratio (RR) with 95% confidence intervals (CI), absolute risk reduction (ARR) with 95% CI, and number needed to treat (NNT) with 95% CI.

Pearson or Spearman correlation coefficients were employed to evaluate the relationships between radiographic and psychosocial measures. Multiple linear regression was used to identify determinants of follow-up PedsQL total score; predictors included follow-up BDI, follow-up SAQ, final follow-up Cobb angle, and treatment group. Logistic regression was used to identify factors associated with curve progression, reporting odds ratios (OR) with 95% CI. Because curve progression was not a rare outcome, ORs are presented as measures of association and should not be interpreted as risk ratios. All tests were two-sided, and *p* < 0.05 was considered statistically significant.

Given the modest cohort size and the limited set of uniformly captured baseline covariates relevant to treatment allocation, we prioritized parsimonious multivariable adjustment and transparent reporting of effect sizes. Baseline patient-reported outcome scores were not included as covariates because longitudinal between-group comparisons were primarily assessed using the group × time mixed ANOVA framework, and the regression analyses were intended to explore cross-sectional associations at follow-up.

Given the number of secondary endpoints, analyses of sagittal parameters and psychosocial outcomes were interpreted as exploratory; we emphasize effect sizes and confidence intervals rather than statistical significance alone. Primary analyses were performed as complete-case analyses for each outcome.

No a priori sample size calculation was performed because of the retrospective design. With 60 participants per group, the study has approximately 80% power (two-sided α = 0.05) to detect a standardized between-group difference in Cohen’s d = 0.52 for continuous outcomes.

### 2.9. Ethical Approval and Consent

The study was conducted in accordance with the Declaration of Helsinki and approved by the Education Planning Committee of Metin Sabancı Baltalimanı Bone Diseases Training and Research Hospital, affiliated with the University of Health Sciences (protocol code: 302; meeting no: 39; approval date: 6 August 2025). Informed consent was obtained as required by the institutional review board; all data were anonymized prior to analysis.

## 3. Results

### 3.1. Participant Characteristics

A total of 120 girls were included 60 treated with TLSO bracing and 60 managed with observation alone. Baseline age was similar between groups (13.6 ± 1.4 vs. 13.7 ± 1.5 years; *p* = 0.74). All patients were skeletally immature (Risser 0–1), with a comparable distribution of Risser grade between groups (Table 1). Mean follow-up duration was approximately 24 ± 6 months in both groups.

### 3.2. Primary Outcome: Curve Progression and Coronal Radiographic Changes

Baseline major curve Cobb angle did not differ significantly between groups (28.0° ± 7.1° in the bracing group vs. 26.5° ± 6.5° in the observation group; *p* = 0.20). At follow-up, the observation group had a higher mean Cobb angle than the bracing group (33.8° ± 12.1° vs. 29.2° ± 9.3°; *p* = 0.03; mean difference −4.6°, 95% CI −8.5 to −0.7; Cohen’s d = −0.43). Mean Cobb angle change was +1.2° with bracing and +7.3° with observation, with a significant group × time interaction (*p* < 0.001; partial η^2^ = 0.20) (Table 2).

Using the predefined progression criterion (ΔCobb ≥ 5° and/or Cobb > 40°), progression occurred in 15% of braced patients (9/60) and 45% of observed patients (27/60). This corresponds to a risk ratio of 0.33 (95% CI 0.17–0.65), an absolute risk reduction of 30.0% (95% CI 14.5–45.5), and a number needed to treat of 4 (95% CI 3–7) to prevent one progression event over approximately two years. Eleven observed patients (18.3%) exceeded 40° during follow-up versus none in the bracing group (Fisher’s exact test *p* < 0.001; absolute risk reduction 18.3%, 95% CI 8.5–28.1; number needed to treat 6, 95% CI 4–12).

### 3.3. Sagittal Spinopelvic Alignment

Across follow-up, sagittal spinopelvic parameters (PT, SS, TK, LL, SPA, and SSA) did not demonstrate statistically significant between-group differences over time (all group × time interaction *p* > 0.05). Thoracic kyphosis showed a non-significant trend toward a group × time difference (*p* = 0.07) (Table 2). Between-group differences at follow-up were small; the mean difference (brace minus observation) was −1.3° for pelvic tilt (95% CI −3.0 to 0.4) and +1.5° for thoracic kyphosis (95% CI −1.9 to 4.9).

### 3.4. Patient-Reported Outcomes

At baseline and follow-up, no statistically significant between-group differences were observed in SRS-22r total score (*p* = 0.96), PedsQL total score (*p* = 0.40), SAQ total score (*p* = 0.78), TAPS score (*p* > 0.05), or BDI score (*p* = 0.67) (Table 3). Effect sizes for between-group differences at follow-up were small (Cohen’s d range: −0.10 to 0.13). For example, the follow-up mean difference (brace minus observation) was −1.0 points for PedsQL (95% CI −4.53 to 2.53) and 0.00 for SRS-22r (95% CI −0.14 to 0.14).

In subgroup analyses, patients who experienced curve progression reported worse appearance-related outcomes than those who remained stable. Specifically, SRS-22r appearance subscores were lower in progressive versus non-progressive patients (3.4 ± 0.5 vs. 4.1 ± 0.4; *p* < 0.01). Higher Cobb angles were associated with worse perceived appearance on SAQ (Cobb > 35° vs. < 30°: 3.35 ± 0.6 vs. 2.55 ± 0.5; *p* < 0.01).

### 3.5. Associations Between Radiographic and Psychosocial Measures and Regression Analyses

Correlation analyses showed that increasing curve magnitude was associated with worse body image perception (higher SAQ) and worse trunk appearance perception (lower TAPS). Higher SAQ scores were associated with lower quality of life scores (SRS-22r and PedsQL). Depressive symptoms (BDI) showed strong associations with both quality of life and body image measures. In contrast, sagittal spinopelvic parameters did not show meaningful correlations with psychosocial measures.

In multiple linear regression assessing determinants of generic quality of life (PedsQL), depressive symptoms were the strongest predictor (standardized β = −0.55, *p* < 0.001), followed by body image concerns (SAQ; standardized β = −0.32, *p* = 0.002). The Cobb angle showed a borderline association (standardized β = −0.18, *p* = 0.054), while the treatment group was not independently associated with PedsQL (standardized β = −0.05, *p* = 0.48). The model explained 52% of the variance in PedsQL (R^2^ = 0.52; F(4,115) = 30.2; *p* < 0.001) (Table 4).

In logistic regression evaluating progression risk, bracing was associated with reduced odds of progression (OR = 0.28; 95% CI 0.10–0.80; *p* = 0.018). Lower skeletal maturity (Risser 0 vs. 1) and higher baseline Cobb angle were associated with increased odds of progression (Table 5).

### 3.6. Brace Adherence

Brace adherence categories were available from clinical records. Adherence was predominantly high (≥16 h/day; 57/60, 95.0%), with only three patients categorized as intermediate (8–16 h/day; 3/60, 5.0%) and none categorized as low adherence (<8 h/day). Accordingly, adherence-related analyses were descriptive, and no hypothesis testing across adherence strata was performed.

## 4. Discussion

This study evaluated spinopelvic alignment together with quality of life, body image, and psychological well-being in adolescent girls with idiopathic scoliosis managed with bracing or observation. The primary finding was that brace treatment significantly reduced curve progression, whereas sagittal spinopelvic parameters remained stable and comparable between groups. Despite the mechanical burden of brace wear, no statistically meaningful differences were observed between the groups in health-related quality of life, body image perception, or depressive symptoms. These findings suggest that, within this retrospective cohort, bracing was not associated with large differences in psychosocial patient-reported outcomes compared with observation; however, because treatment allocation was not randomized and brace wear was self-reported, psychosocial comparisons should be interpreted cautiously.

Importantly, depression explained more variance in quality of life than any radiographic parameter, including Cobb angle, underscoring that mental health status outweighs curve magnitude in determining patient-reported outcomes. This highlights that in adolescent idiopathic scoliosis, the patient’s psychological response to the deformity is more decisive than the anatomical severity of the curve itself. While brace treatment effectively controls structural progression, its impact on daily functioning and well-being is largely mediated by emotional and perceptual factors rather than radiographic change. These results reinforce the need for a biopsychosocial approach in AIS management, integrating psychological screening and support alongside orthopedic treatment.

In our study, the Cobb angle increased by an average of 1.2° in the brace group and 7.3° in the observation group (*p* < 0.001). 70% of the brace group was stable, and improvement was observed in 15%. These rates are consistent with the 73.2% success rate in the meta-analysis of Costa et al. [5]. Babaee et al. also showed that the brace controlled the progression in curvatures above 40° [17]. In our logistic regression model, brace treatment was associated with lower odds of progression (OR = 0.28), whereas lower skeletal maturity (Risser grade 0 vs. grade 1) and a higher baseline Cobb angle were associated with higher odds of progression (Table 4). The research by Chan et al. includes a meta-analysis, in which the chance of success was found to be 3.58 times higher in those with a Cobb angle below 30°, and it was emphasized that full-time compliance increased success by 5.22 times [18]. The research showed that Risser stage 0–1 patients faced a 3.5 times higher chance of disease advancement. The study results showed that each 1° increase in the initial Cobb angle measurement would lead to an 8% higher risk of disease progression. The research results confirm the results of Babaee et al. The research showed that patients who had not finished their bone growth process faced the highest risk [17]. Costa et al. also reported that the brace was most effective in Risser 0–2 and 0–3 stages [5]. In our observation group, 18% of patients exceeded the surgical threshold of 40°. In the study of Dolan et al., based on BrAIST data, it was reported that only 42% of untreated patients had good results [19]. The regression model in the same study predicts the risk of failure with high accuracy with age, gender, and Risser stage [19].

The research findings showed that thoracic kyphosis measurements decreased from 30.2° to 28.5°, but the difference between these values did not achieve statistical significance (*p* = 0.07). Zhang et al.’s research showed that patients who wore braces for an extended period experienced a decrease in their thoracic kyphosis measurements from 24.35° to 19.02° [20]. In the review of Ghorbani et al., it was emphasized that braces flatten thoracic kyphosis and lumbar lordosis [15]. The research showed that lumbar lordosis measurements decreased from 51.0° to 49.0°, but the change was not statistically significant (*p* > 0.1); within the follow-up period, we did not observe statistically significant reductions in lumbar lordosis, suggesting no evidence of clinically relevant sagittal flattening attributable to bracing in this cohort. Pelvic incidence remained stable as an anatomical parameter; there was no significant difference between the groups in pelvic tilt and sacral slope. In our cohort, pelvic incidence and lumbar lordosis showed a positive correlation of r = 0.60 at *p* < 0.001. The study results showed that pelvic compensation mechanisms operate correctly. There was no significant relationship between spinopelvic parameters and psychosocial measures (|r| < 0.2). Although Zhou et al. showed that the PI–LL difference in degenerative scoliosis affects quality of life [21], this difference is thought to be most likely population-dependent; while body image is more related to cosmetic appearance in adolescents, pain and functional limitation are at the forefront in degenerative patients.

We did not find a significant difference in quality of life scores between the brace and observation groups (SRS-22r, *p* = 0.96; PedsQL *p* = 0.40). Taken together, these findings suggest that, in this observational cohort, brace treatment was not associated with a large additional psychosocial burden relative to observation alone; nevertheless, unmeasured differences in baseline illness perception, motivation, coping, and family support between families choosing bracing versus observation may have influenced patient-reported outcomes. The SRS-22r appearance score showed that patients who had their condition had different results than those who did not (3.4 vs. 4.1; *p* < 0.01). Belli et al.’s study [22] showed that scoliosis disease progression leads to negative impacts on physical appearance and life quality for all scoliosis patients at any disease severity level. The SAQ 3.35 score for patients with Cobb angles greater than 35° was significantly higher than the 2.55 score for patients with lower Cobb angles (*p* < 0.01). Kinel et al.’s research, which was conducted by [23], showed that patients who had Cobb angles greater than 30° developed worsening quality of life. Zaina et al. showed that body image concerns became more evident as curvature increased [24]. These findings indicate that curve control and perceived deformity drive the quality of life more than treatment modality. In descriptive analyses, satisfaction tended to be higher among patients reporting high brace wear (4.0 vs. 3.2); however, adherence variability was limited and self-reported, precluding robust dose–response analyses. Feelings of negative body image, according to Bertuccelli and colleagues, tend to lower quality of life while also making it harder to find well-fitting braces, a point they emphasized [25].

The research results showed that depression created the biggest negative effect on life quality (β = −0.55). In the review of Mitsiaki et al., it was emphasized that depressive symptoms have a strong effect on the quality of life in individuals with ILS and that this is more pronounced in female adolescents [26]. Body image emerged as the second most influential factor, which determined the outcome at β = −0.32, while the Cobb angle measurement showed a weak association (β = −0.18; *p* = 0.054). In the multicenter study of Bagó et al., a weak relationship was found between curvature size and pain, and psychosocial factors such as anxiety and depression were more decisive [27]. Kerr et al.’s study also demonstrated that postoperative quality of life depends most heavily on catastrophizing and anxiety [28]. The research shows body shape influences life quality because it influences body image perception and mental wellness. Mitsiaki et al. support the observed relationship between body image and depression, which shows a positive correlation of r = +0.40 [26]. Our correlation between SAQ and SRS-22r outlook subdimension (r = −0.60) is very close to the values reported in the same study (r = −0.58). The type of treatment had no direct effect on quality of life (β = −0.05); this result is parallel to the study of Cheung et al. [8]. The study by Limbers et al. established that young women who experience body image anxiety develop eating disorders, which negatively impact their quality of life [29]. The research findings show that psychological screening functions as a critical assessment tool that enables patients to improve their quality of life while they follow their medical treatment.

This study has limitations inherent to its retrospective, non-randomized design. First, confounding by indication and selection bias are possible, as treatment allocation was determined by routine clinical decision-making and shared decision-making rather than randomization. Importantly, the decision to initiate bracing may be associated with baseline illness perception, motivation, coping style, and family support, which could particularly confound psychosocial outcomes and are difficult to quantify retrospectively. Although baseline characteristics were comparable and regression models adjusted for baseline Cobb angle and skeletal maturity, residual confounding from unmeasured factors (e.g., curve pattern, menarchal status, growth velocity, family preference, and psychosocial support) cannot be excluded; more advanced causal-inference approaches (propensity score methods) may be informative in larger datasets with richer baseline covariate measurement. Second, brace adherence was based on patient/parent report and clinical documentation; objective monitoring was not available. The near-uniformly high adherence distribution may overestimate the true brace dose, potentially inflating apparent brace effectiveness and precluding meaningful dose–response analyses. Third, the follow-up duration (24 ± 6 months) captures clinically relevant short- to mid-term progression risk but may be insufficient to evaluate longer-term sagittal profile changes and sustained psychosocial trajectories after brace discontinuation; additionally, because sagittal parameters were secondary outcomes, the study may be underpowered to detect small between-group differences. Finally, while we did not detect between-group differences in psychosocial outcomes, the study was not designed as an equivalence or non-inferiority trial; therefore, small differences in patient-related outcomes cannot be definitively excluded. Key strengths include a relatively homogeneous cohort restricted to skeletally immature girls with moderate AIS, standardized radiographic acquisition with a defined out-of-brace interval at follow-up, and the simultaneous evaluation of multiple validated psychosocial instruments alongside radiographic and spinopelvic parameters. In addition to statistical testing, we report clinically interpretable risk measures (RR, absolute risk reduction, and NNT) for the primary endpoint.

Prospective studies incorporating objective brace-wear monitoring, longer follow-up beyond brace discontinuation, and psychologically informed assessments (illness perception, coping, and family support), as well as analytical approaches such as propensity score methods, could further strengthen causal inference regarding the psychosocial and sagittal effects of bracing. Integrating routine mental health screening into AIS pathways should also be evaluated as a strategy to improve patient-centered outcomes.

## 5. Conclusions

In this retrospective comparative cohort of skeletally immature girls with moderate AIS, TLSO bracing was associated with a substantially lower risk of curve progression over approximately two years compared with observation. Under a standardized out-of-brace radiographic protocol, we did not observe clinically relevant between-group differences in sagittal spinopelvic alignment or multidomain psychosocial PROs. Nevertheless, due to non-randomized allocation and self-reported brace wear, psychosocial comparisons should be interpreted cautiously and confirmed in prospective studies with objective adherence monitoring and psychologically informed assessments. Across measures, curve progression and depressive symptoms were more strongly associated with patient-reported well-being than treatment modality, supporting the value of integrating psychosocial screening into AIS follow-up.

## Figures and Tables

**Table 1:** Baseline demographic and clinical characteristics.

Variable	Brace Group (*n* = 60)	Observation Group (*n* = 60)	*p* Value
Age, years	13.6 ± 1.4	13.7 ± 1.5	0.74
Risser grade 0, *n* (%)	24 (40.0)	19 (31.7)	0.34
Risser grade 1, *n* (%)	36 (60.0)	41 (68.3)	
Baseline major curve Cobb angle, °	28.0 ± 7.1	26.5 ± 6.5	0.20
Follow-up duration, months	24 ± 6	24 ± 6	1.00 †
Brace wear duration, months	20 (mean)	—	—

† Equal follow-up duration by design; *p* value reflects identical mean follow-up in both groups. —: not applicable.

**Table 2:** Radiographic outcomes and curve progression over follow-up.

Parameter	Brace Baseline	Brace Follow-Up	Observation Baseline	Observation Follow-Up	Change (Δ, Follow-Up–Baseline)	*p* Value (Group × Time Interaction)
**Coronal plane**						
Major curve Cobb angle, °	28.0 ± 7.1	29.2 ± 9.3	26.5 ± 6.5	33.8 ± 12.1	+1.2 (*p* = 0.40)/+7.3 (*p* < 0.001)	<0.001
Effect size (Cobb angle group × time)	Partial η^2^ = 0.20; Cohen’s d = 0.7					
**Sagittal spinopelvic parameters, °**						
Pelvic incidence (PI) ‡	47.8 ± 8.5	—	48.5 ± 7.9	—	—	NA
Pelvic tilt (PT)	9.8 ± 4.2	10.5 ± 4.5	9.5 ± 4.0	11.8 ± 4.8	+0.7/+2.3	0.25
Sacral slope (SS)	38.0 ± 7.8	37.3 ± 7.5	39.0 ± 7.5	36.7 ± 7.2	−0.7/−2.3	>0.05
Thoracic kyphosis (TK)	30.2 ± 10.0	28.5 ± 9.0	25.4 ± 11.0	27.0 ± 10.0	−1.7/+1.6	0.07
Lumbar lordosis (LL)	51.0 ± 12.0	49.0 ± 13.0	50.5 ± 11.0	51.2 ± 12.0	−2.0/+0.7	>0.10
Spinopelvic angle (SPA)	131.4 ± 8.2	130.9 ± 8.0	132.0 ± 7.8	131.0 ± 7.6	−0.5/−1.0	>0.50
Spinosacral angle (SSA)	130.2 ± 7.5	129.5 ± 7.3	131.0 ± 7.2	130.2 ± 7.0	−0.7/−0.8	>0.05
**Curve status at follow-up (based on ΔCobb)**						
Improved (ΔCobb ≤ −5°), *n* (%)	9 (15.0)		0 (0.0)			0.003 §
Stable (|ΔCobb| < 5°), *n* (%)	42 (70.0)		33 (55.0)			0.13 ¶
Progression (ΔCobb ≥ +5°), *n* (%)	9 (15.0)		27 (45.0)			<0.001 ¶
Exceeded 40° at follow-up, *n* (%)	0 (0.0)		11 (18.3)			<0.001 §

Δ indicates follow-up minus baseline. *p* values in the last column are from two-way mixed ANOVA (group × time interaction) for continuous radiographic parameters. For the Cobb angle, within-group paired *t* test *p* values are shown in the Change column. Categorical curve-status comparisons use chi-square or Fisher’s exact test as appropriate. ‡ Pelvic incidence is an anatomical parameter (PI = PT + SS) and is reported at baseline. § Fisher’s exact test. ¶ Chi-square test. —: not applicable. Exact *p* values for nonsignificant interactions should be reported (replace ‘>0.05’ with exact values if available).

**Table 3:** Patient-reported outcomes, subgroup analyses, and brace adherence.

Outcome	Brace Baseline	Brace Follow-Up	Observation Baseline	Observation Follow-Up	*p* Value (Group Main Effect)	*p* Value (Group × Time Interaction)
**Longitudinal patient-reported outcomes**						
SRS-22r total score	4.0 ± 0.4	3.9 ± 0.4	4.1 ± 0.3	3.9 ± 0.4	0.96	>0.05 *
PedsQL total score	85.2 ± 9.8	83.0 ± 10.2	86.5 ± 8.7	84.0 ± 9.5	0.40	>0.05 *
SAQ total score †	2.85 ± 0.5	2.95 ± 0.5	2.78 ± 0.6	2.88 ± 0.6	0.78	0.88
TAPS score ‡	3.52 ± 0.6	3.45 ± 0.6	3.58 ± 0.5	3.50 ± 0.5	>0.05	>0.05 *
BDI score	8.2 ± 5.0	9.0 ± 6.0	7.5 ± 4.8	8.4 ± 5.5	0.67	>0.05 *
**Subgroup/exploratory analyses (cross-sectional)**						
SRS-22r appearance subscore (progressive vs. non-progressive) §	Progressive: 3.4 ± 0.5; Stable: 4.1 ± 0.4				—	<0.01
SAQ subjective appearance (Cobb > 35° vs. <30°) ¶	Cobb > 35°: 3.35 ± 0.6; Cobb < 30°: 2.55 ± 0.5				—	<0.01
**Brace adherence (brace group only; exploratory)**						
<8 h/day (low)	0 (0.0)	SRS-22r satisfaction	—		—	—
8–16 h/day (intermed.)	3 (5.0)	SRS-22r satisfaction: 3.2 ± 0.6	—		—	—
≥16 h/day (high)	57 (95.0)	SRS-22r satisfaction: 4.0 ± 0.4	—		—	—

* For outcomes where the interaction *p* value was not explicitly reported in the original table, values are indicated as >0.05 based on the stated mixed-ANOVA results. † Higher SAQ scores indicate worse perceived appearance. ‡ Higher TAPS scores indicate better perceived appearance. § Progression was defined as ΔCobb ≥ 5° and/or Cobb angle > 40° at follow-up; subgroup comparisons were performed between progressive (*n* = 36) and non-progressive patients (*n* = 84). ¶ Subgroup comparisons by Cobb angle category were performed using appropriate parametric or non-parametric tests depending on distribution. —: not applicable.

**Table 4:** Multiple linear regression for determinants of PedsQL total score.

Predictor/Variable	Standardized β	*p* Value
Model summary	R^2^ = 0.52; F(4,115) = 30.2	*p* < 0.001
BDI (per 1 point)	−0.55	*p* < 0.001
SAQ total score (per 1 unit)	−0.32	0.002
Cobb angle at final follow-up (per 1°)	−0.18	0.054
Treatment group (brace vs. observation)	−0.05	0.48

Standardized β coefficients are shown to enable comparison across predictors. BDI: Beck Depression Inventory; SAQ: Spinal Appearance Questionnaire; PedsQL: Pediatric Quality of Life Inventory.

**Table 5:** Logistic regression for predictors of curve progression.

Predictor/Variable	OR	95% CI	*p* Value
Model summary	Nagelkerke R^2^ = 0.35; χ^2^(3) = 29.4		*p* < 0.001
Brace treatment (reference: observation)	0.28	0.10–0.80	0.018
Risser grade 0 (reference: grade 1)	3.50	1.45–8.45	0.007
Baseline Cobb angle (per 1°)	1.08	1.01–1.16	0.044

Progression was defined as ΔCobb ≥ 5° and/or Cobb > 40°. OR: odds ratio; CI: confidence interval.

## Data Availability

The datasets generated and/or analyzed during the current study are available from the corresponding author upon reasonable request due to ethical and privacy restrictions.

## Abbreviations

PI: Pelvic Incidence | PT: Pelvic Tilt | SS: Sacral Slope | TK: Thoracic Kyphosis | LL: Lumbar Lordosis | SSA: Spinosacral Angle | SPA: Spinopelvic Angle | SRS-22r: Scoliosis Research Society-22 | PedsQL: Pediatric Quality of Life Inventory | SAQ: Spinal (Scoliosis) Appearance Questionnaire | TAPS: The Trunk Appearance Perception Scale | BDI: Beck Depression Inventory | OR: Odds Ratio | CI: Confidence Interval.
